# Mississippi Valley Association of Dental Surgeons

**Published:** 1846-12

**Authors:** 


*Mississippi Valley Association of Dental Surgeons.*—We have received a pamphlet containing the proceedings of the second annual meeting of the above named association, from which we infer, that it was very well attended. Among the matters which came up for discussion, the subject of patenting improvements in instruments, dental appliances and modes of practice, seems to have been the most exciting. This subject, however, after having been pretty fully debated, was laid over for subsequent action of the society. We also observe, in the proceedings, a resolution condemnatory of the practice, which too many members of the profession are guilty of, of setting forth in advertisements, their own peculiar merits, or proposing to render their services for less compensation than is usually charged. The resolution, we are gratified to see, makes this, on the part of the members of the association, an offence rendering any who may be guilty of it, liable to expulsion. A resort to such degrading means as this for the procurement of practice should not be countenanced by the members of the profession any where.

It would seem from the printed proceedings that the claim of Dr. Allen to the authorship of a dental improvement, for which he had obtained letters patent—was called in question by some member of the association, and with a view of ascertaining the justness of said claim, a committee was appointed to investigate the matter. After having done this, it reported that there was no reason to believe that he was not the author of the improvement.

The propriety of publishing a quarterly journal devoted to the interests of the profession was also discussed at the meeting, and a committee appointed to ascertain the cost, with instructions to issue, at their discretion, a specimen number during the coming year at an expense not exceeding fifty dollars, or merely to publish a prospectus, as they might think proper. We hope our brethren of the Mississippi Valley Association may be successful in this proposed undertaking, but we fear the difficulties they will experience in carrying it out, will prevent them from accomplishing it at this time. Very few, comparatively, of the members of the dental profession are reading men, and what is worse, they do not care to become so. Hence the great difficulty of sustaining publications of this sort. But we would not say any thing to discourage our friends of the West, nor those of the Virginia Society, who by the by, as we perceive from the proceedings of their last meeting, have in view a similar undertaking; on the contrary, we shall most cheerfully use what little influence we may possess, to extend the circulation of both publications, should they be started. We fear, however, that neither will meet with the encouragement, which, we have no doubt they will deserve.

The officers elected for the ensuing year are, Drs. Ed. Taylor, president; W. E. Ide, M. Rogers, and A. Berry, vice-presidents; W. B. Ross, recording secretary; James Taylor, corresponding secretary; C. Bonsal, treasurer, and H. Crane, J. Allen and J. W. Cook, examining committee.

We should like to notice the proceedings more at length, but time will not permit us to do so at present.—*Balt. Ed.*

